# Gut microbiome signatures associated with depression and obesity

**DOI:** 10.1128/msystems.01263-25

**Published:** 2026-01-30

**Authors:** Carlos Mora-Martínez, Gara Molina-Mendoza, María Carmen Cenit, Eva M. Medina-Rodríguez, Ana Larroya-García, Yolanda Sanchez-Carro, Leticia Gonzalez-Blanco, Julio Bobes, Pilar Lopez-Garcia, Mercedes Zandio-Zorrilla, Francisca Lahortiga-Ramos, Margalida Gili, Mauro Garcia-Toro, Bernardino Barcelo, Olga Ibarra, Yolanda Sanz

**Affiliations:** 1 Microbiome Innovation in Nutrition and Health Unit, Institute of Agrochemistry and Food Technology, National Research Council (IATA-CSIC), Valencia, Spain; 2 Psychiatry Service, Doctor Peset University Hospital, FISABIO 203353 , Valencia, Spain; 3 Department of Psychology, Universidad Europea de Canarias, Canarias, Spain; 4 Department of Psychiatry, University of Oviedo, Centro de Investigación Biomédica en Red de Salud Mental, CIBERSAM 16763, Oviedo, Spain; 5 Center for Biomedical Research in Mental Health (CIBERSAM), Carlos III Health Institute 38176, Madrid, Spain; 6 Department of Psychiatry, Universidad Autonoma de Madrid (UAM) 16722, Madrid, Spain; 7 Department of Psychiatry, Instituto de Investigación Sanitaria Princesa (IIS-IP), Madrid, Spain; 8 Navarra Institute for Health Research (IdiSNA), Pamplona, Spain; 9 Department of Psychiatry and Medical Psychology, University Clinic of Navarra 16755, Pamplona, Spain; 10 Health Research Institute of the Balearic Islands (IdISBa), Palma, Spain; 11 University Institute of Health Science Research (IUNICS), University of the Balearic Islands (UIB) 16745 , Palma, Spain; 12 Department of Medicine, University of the Balearic Islands 16745 , Palma, Spain; 13 Clinical Analysis Service, Clinical Toxicology Unit, Hospital Universitario Son Espases 375118, Palma de Mallorca, Spain; Northern Arizona University, Flagstaff, Arizona, USA

**Keywords:** metagenomics, depression, major depressive disorder, body mass index, *Faecalibacterium prausnitzii*, overweight, machine learning

## Abstract

Depression and obesity are highly comorbid and likely involve common risk factors and pathophysiological mechanisms, which could crosslink to gut microbiome dysfunction. Here, we performed a case-control study with a total of 105 subjects, 43 with major depressive disorder (MDD) and 62 non-depressed controls free from psychiatric comorbidities, to identify gut microbiome signatures associated with MDD and dissect its relation to body mass index (BMI) and lifestyle (diet and exercise). We performed shotgun metagenomics, followed by taxonomic and functional annotations. Using different machine learning methods, we were able to classify subjects into depressed and non-depressed controls with a balanced accuracy of 0.90 and into depressed or non-depressed and normal weight or overweight with a balanced accuracy of 0.78 based solely on taxonomic profiles. We identify novel bacterial taxa associated with depression, including reductions in *Butyrivibrio hungatei* and *Anaerocolumna sedimenticola,* and also replicate previously reported associations, such as decreased *Faecalibacterium prausnitzii* in patients with MDD. Functional annotation of metagenomes shows differences in pathways linked to the synthesis of fundamental nutrients, which have been associated with diet, as well as inflammation. Strikingly, we found an increase in tryptophan degradation and a decrease in queuosine synthesis pathways, both of which are directly related to a decrease in monoaminergic neurotransmitter availability. Additionally, our functional analysis shows that most of the functions that are more abundant in controls than in depressed subjects are encoded by *F. prausnitzii*. These findings reveal distinct microbial and functional signatures associated with depression, including taxa and pathways linked to neurotransmitter metabolism and independent of other covariates. This suggests that gut microbiome profiling could support diagnosis and the development of gut-directed depression treatments.

**IMPORTANCE:**

This study identifies gut microbiome signatures that are predictive of major depressive disorder (MDD) and explores their links to body mass index (BMI). We uncover bacterial species and metabolic pathways that are associated with MDD, some of them related to neurotransmitter metabolism and inflammation. Among the differences identified, depletion of *Faecalibacterium prausnitzii* stands out as an important feature in the MDD microbiome, which suggests the possible use of this species to improve depression symptoms. Importantly, we demonstrate shared microbiome features between MDD and BMI, suggesting common underlying mechanisms. This research not only provides a framework for developing microbiome-based diagnostics but also informs future stratified interventions targeting gut microbial functions to improve mental health outcomes.

## INTRODUCTION

Mental health is a fundamental human right and, according to the World Health Organization, is critical for personal, community, and socioeconomic development. Epidemiological data from 2019 showed that approximately 970 million people struggled with a mental health disorder (one in eight people), with anxiety and depressive disorders being the most prevalent worldwide (1). In 2020, the number of people with depressive disorders increased by 28% due to the global impact of the COVID-19 pandemic (Global Health Data Exchange, GHDx) (2, 3). After the pandemic peak, many countries, such as the United Kingdom, the United States, and Korea, show slight decreases in symptoms of depression, but the prevalence remains at least 20% higher than that prepandemic, according to the Organisation for Economic Co-operation and Development (OECD) (4). A recent repeated cross-sectional study conducted in Finland, involving more than 150,000 students aged 13–20 years, showed that the proportion of participants with generalized anxiety remained equally high in 2023 as in 2021, with an increase in girls (5). This suggests that the effects of the pandemic on mental health could be long-lasting.

Major depressive disorder (MDD) is a debilitating illness characterized by low mood, loss of interest, impaired cognitive function, and vegetative symptoms such as disturbed sleep or appetite (6). Common treatments include psychological interventions, such as cognitive-behavioral therapy, and pharmacological treatments, primarily involving selective serotonin reuptake inhibitors (SSRIs), noradrenaline reuptake inhibitors (NRIs), serotonin and noradrenaline reuptake inhibitors (SNRIs), noradrenaline and dopamine reuptake inhibitors (NDRIs), and tricyclic antidepressants (TCAs), sometimes combined with antipsychotics (7–9). However, more than one-third of patients do not respond adequately, and clinical improvement often takes several weeks (10–12). More recently, intranasal esketamine has shown faster effects and promising outcomes in treatment-resistant depression (13).

Depression has been consistently linked to obesity and, together with obesity-related metabolic disorders, these pathologies show high rates of comorbidity, reducing the efficacy of the treatments (14–16). Accordingly, these conditions may share common risk factors or pathogenic mechanisms, some of which may be driven by a dysfunctional gut microbiome. A better understanding of the differential and shared microbiome characteristics of individuals with depression with and without comorbid obesity is, therefore, crucial to elucidate the potential underlying causes and to better categorize patients and tailor treatments (16).

The gut microbiome may influence mood through a network of bidirectional connections within the gut-brain axis. Indeed, a number of studies have described the differences in microbial composition between patients with MDD and healthy individuals, both in terms of microbial diversity and the relative abundance of several bacterial taxa (17–23). For instance, several studies have reported an increase in the Bacteroidota/Bacillota (formerly named Bacteroidetes/Firmicutes) ratio in patients with MDD, characterized by an enrichment of the genus *Bacteroides* and a depletion of the genera *Blautia*, *Faecalibacterium*, *Ruminococcus, Subdoligranulum,* and *Coprococcus* (18, 19, 21, 22, 24–30). These genera are known producers of short-chain fatty acids such as butyrate, which have neuroprotective and anti-inflammatory roles (19, 31–34). Moreover, different studies have demonstrated an increase in *Eggerthella* (18, 21, 23, 25, 28, 35, 36), an Actinobacteria linked to pro-inflammatory metabolism and disturbed neurotransmitter processing (37), *Streptococcus* (Firmicutes) (21, 22, 25, 28, 29, 36), and *Parabacteroides* (Bacteroidota) (21, 25, 26, 28, 29) and a decrease in *Sutterella* (22, 28, 35, 36), a genus potentially involved in mucosal immunity and gut epithelial regulation (38, 39). Some studies have also reported *Dialister* (19, 23, 26, 28, 29) and *Prevotella* (23, 25, 26, 40–42) to be depleted in depression. However, the results are sometimes conflicting regarding these groups (22, 28), and some studies also associate *Dialister* and *Prevotella* with inflammation and other negative health outcomes (43, 44). Therefore, their functional role needs further study. Disturbances in the gut microbiome, including a reduction in diversity, have also been linked to higher body mass index (BMI) and related obesity-associated metabolic perturbations (45–47).

Many more associations have been reported, but these findings are often inconsistent due to the high interpersonal variability, the lack of control for the numerous variables that influence the gut microbiome (e.g., diet, exercise, medications, and comorbid conditions), and differences in the methods used for the analysis (19, 29, 48). In particular, the potential role of the gut microbiome in depression comorbid with obesity is largely understudied, although data suggest that a combined approach of monitoring and targeting the gut microbiome may prove more efficacious in treating their comorbidity (15).

Additionally, antidepressants interact in complex ways with the gut microbiome (49–52). *In vitro* studies have shown antimicrobial effects of compounds like fluoxetine against species such as *Lactobacillus rhamnosus*, *Ligilactobacillus salivarius*, *Escherichia coli*, or *Enterococcus faecalis* (53–55). Animal studies report changes in alpha and beta diversity, as well as in specific taxa (53, 56). In humans, some studies associate antidepressant use with microbiome shifts (57, 58), while others report no significant effects (30, 59). A recent meta-analysis suggests that antidepressants may not cause broad alterations in diversity but are consistently associated with changes in the abundance of 19 specific genera across multiple studies (50). Conversely, microbiome composition may influence antidepressant efficacy, and differences have been observed between treatment-resistant and -responsive patients (49, 60).

The majority of studies linking microbiome and depression employ low-resolution analytical techniques (short 16S rRNA gene amplicons) (18). While some amplicon sequence variants (ASVs) can be resolved at the species level, this depends on the specific hypervariable region sequenced and the specific algorithms used for clustering sequences and classification. In many cases, resolution is limited to the genus level. Moreover, 16S-based approaches do not directly capture the functional or metabolic potential of the microbiome. These limitations have constrained our ability to identify specific bacterial species and their roles in the pathophysiology of depression (61).

Here, we conducted a case-control study of 105 subjects and carried out a comprehensive taxonomic and functional assessment of the microbiome to identify bacterial signatures predictive of depression and ascertain their association with BMI. Additionally, we identified microbiome functions that may provide a mechanistic explanation for the observed associations with disease phenotypes. This could contribute to the development of BMI-tailored strategies to treat depression in the near future, thereby advancing the field of precision medicine and improving patients’ mental health.

## RESULTS

### Clinical and demographic characteristics

The demographic and clinical characteristics of the participants are shown in Table 1. The cohort included 43 patients with MDD (60% females), with an average age of 50 years, and 62 controls (56% females), with an average age of 42 years. The proportion of women to men was greater in both groups, although the difference was not significant. The difference in age between both the groups was significant (*P* < 0.01). The prevalence of obesity and overweight was also higher in the MDD group, but the difference was not significant. BMI was significantly higher in the MDD group than in the control group.

**TABLE 1:** Demographic and clinical features of MDD patients and controls^*a*^

Parameter	MDD (*n* = 43)	Controls (*n* = 62)	*P*-value^*b*^
Gender (*n =* female)	26 (60%)	35 (56%)	0.8346
Age, years, mean ± SD	50 *±* 10.59	42 *±* 14.05	**0.0088**
Relationship status (*n* = yes)	25 (58%)	50 (82%)	**0.0209**
NA (*n*)	0	1	
Education, degree level			**0.0015**
Primary school (*n*)	4 (10%)	2 (3%)	
Secondary school (*n*)	4 (10%)	0 (0%)	
Technical education (*n*)	20 (48%)	17 (28%)	
University (*n*)	14 (33%)	41 (68%)	
NA (*n*)	1	2	
BMI, kg/m^2^, mean ± SD	26.94 *±* 5.65	24.43 *±* 4.00	**0.0181**
Obesity (*n =* obese plus overweight subjects)	24 (56%)	22 (37%)	0.0842
NA (*n*)	0	2	
Dyslipidemia (*n*)	15 (28%)	8 (15%)	0.6259
NA (*n*)	11	40	
Fasting hyperglycemia (*n*)	1 (3%)	2 (14%)	0.2090
NA (*n*)	11	36	
Type II diabetes (*n*)	4 (10%)	2 (3%)	0.3907
NA (*n*)	1	3	
Hypertension (*n*)	13 (43%)	8 (40%)	0.2090
NA (*n*)	13	42	
Current smoking (*n*)	12 (36%)	8 (13%)	**0.0180**
NA (*n*)	10	1	
Medication (*n*)	42 (91%)	4 (9%)	**<0.0001**
Self-reported alcohol abuse (*n*)	1 (3%)	3 (5%)	1.0000
NA (*n*)	10	1	
Mediterranean diet adherence			0.8102
Low adherence (*n*)	7 (23%)	10 (18%)	
Need improvements (*n*)	18 (60%)	37 (65%)	
Optimal adherence (*n*)	5 (17%)	10 (18%)	
NA (*n*)	13	5	
Physical activity			**<0.0001**
Subject with low IPAQ (*n*)	22 (51%)	6 (10%)	
Moderate IPAQ (*n*)	15 (35%)	30 (49%)	
High IPAQ (*n*)	6 (14%)	25 (41%)	
NA (*n*)	0	1	
High perceived stress, PSS			**<0.0001**
Low (*n*)	0 (0%)	19 (31%)	
Moderate (*n*)	5 (15%)	32 (52%)	
High (*n*)	28 (85%)	10 (16%)	
NA (*n*)	10	1	
Diagnosis			
HDRS-17 score, mean *±* SD	20.22 *±* 5.63	NA	NA
MADRS-10 score, mean *±* SD	27.17 *±* 9.50	NA	NA
BDI mean *±* SD	32.64 *±* 12.75	4.81 *±* 5.70	**<0.0001**
Stool consistency, Bristol stool scale			**0.0237**
Normal transit (*n*)	22 (69%)	48 (91%)	
Diarrhea or constipation (*n*)	10 (31%)	5 (9%)	
NA (*n*)	11	9	

^*a*^ NA, not applicable.

^*b*^ Wilcoxon test for numerical variables, or a χ^2^ test for categorical variables. Bold indicates *P *< 0.05.

In total, 91% of MDD patients took medication compared to only 9% of the controls. Among patients with MDD, the most common medications were SNRIs (*N* = 35), anxiolytics (*N* = 33), anticonvulsants (*N* = 11), proton pump inhibitors (*N* = 10), statins (*N* = 9), beta blockers (N = 7), antipsychotics (N = 7), analgesics (N = 6), hypnotics (N = 6), and antidiabetics (*N* = 3). Among controls, treatments included beta blockers (N = 2), antidiabetics (N = 2), and analgesics (N =2).

The percentage of current smokers was also significantly higher in the MDD group than in the control group, but no differences were observed in alcohol consumption. Finally, the MDD group had a lower, albeit nonsignificant, degree of adherence to the Mediterranean diet than the control group, and physical activity was significantly higher in the control group than in the MDD group.

Patients with MDD were diagnosed at hospitals based on the Diagnostic and Statistical Manual of Mental Disorders (DSM-5). Additionally, patients were stratified according to the severity of their depressive symptoms using the Hamilton Depression Rating Scale (HDRS) and the Montgomery Åsberg Depression Rating Scale (MADRS), which reported scores of 20.22 ± 5.63 and 27.17 ± 9.50 (mean ± SD), respectively. Finally, the self-reported Beck’s Depression Inventory (BDI) was administered to both patients with MDD and non-depressed subjects, with values lower than 10 considered normal, 10–18 mild depression, 19–29 moderate depression, and values higher than 30 severe depression. The MDD group had a significantly higher score than the non-depressed group (32.64 ± 12.7 vs 4.81 ± 5.70, mean ± SD).

### Depressed subjects show distinct patterns of alpha and beta diversity

We obtained between 67.6 and 209 million raw reads per sample, with an average of 140.5 ± 34.6 million. Trimming of low-quality ends and removal of host-derived sequences resulted in 56.2–176.7 million reads per sample, with an average of 114.7 ± 28.7. Of these, an average of 44.3% ± 9.2% were taxonomically assigned using Kraken2. Despite uniform sample processing and sequencing in a single batch, there were significant differences in sequencing depth between patients with MDD and controls (see Table S1; Fig. S1A and B at https://doi.org/10.5281/zenodo.16993542). The source of these differences could be either technical or biological, such as differences in microbial load or in stool composition. Appropriate normalization techniques were applied during the statistical analysis to avoid any bias due to differences in sequencing depth (see Methods and the following sections). Rarefaction analysis indicates saturation of taxa in all samples at minimum sequencing depth (see Fig. S1C at https://doi.org/10.5281/zenodo.16993542).

**Fig 1:**
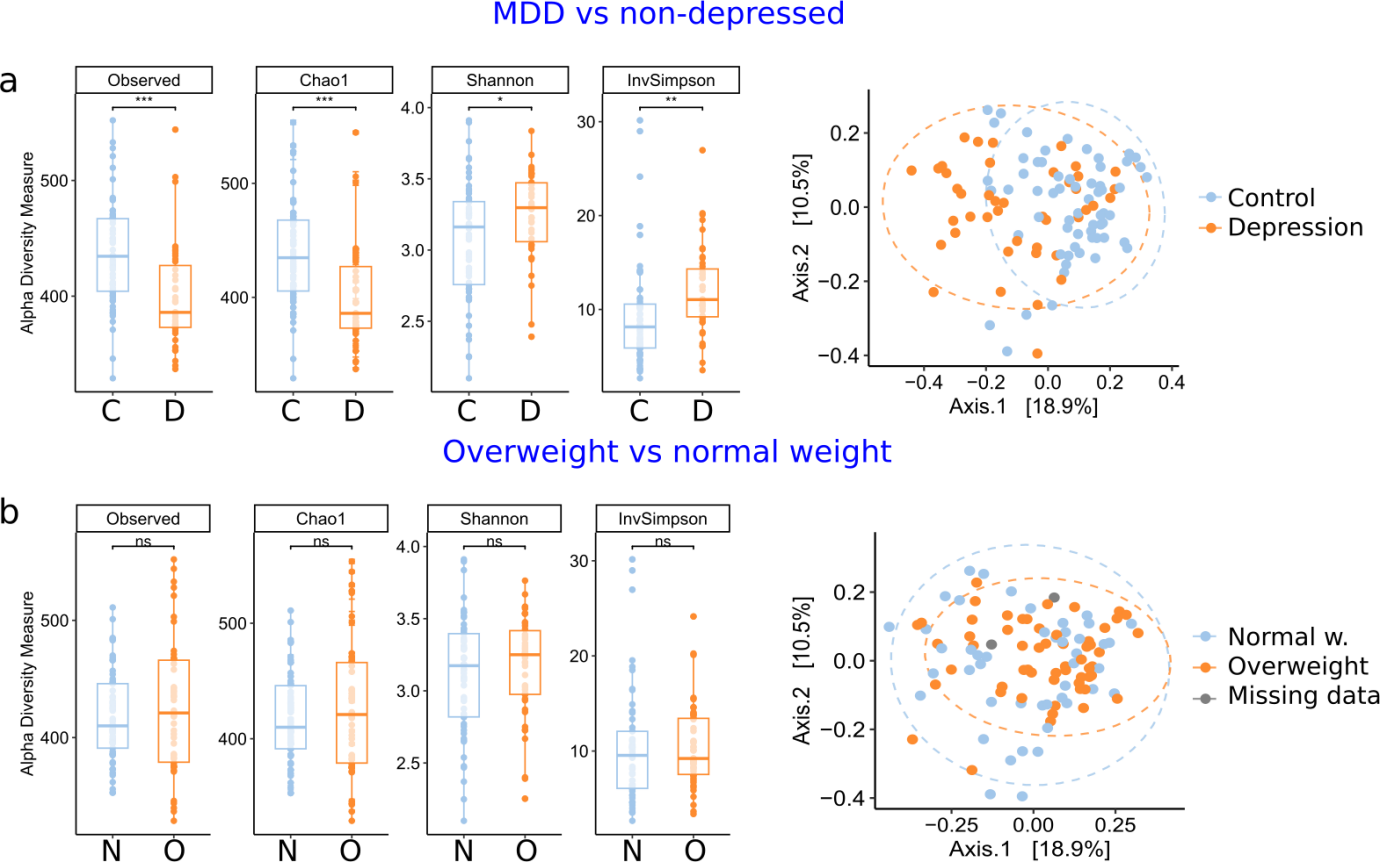
Alpha and beta diversity. (**a**) Differences in alpha and beta diversity between MDD patients and non-depressed subjects. (**b**) Differences in alpha and beta diversity between subjects with normal weight and those with overweight or obesity (BMI >25).

Alpha and beta diversity measures were calculated on abundances rarefied to a minimum depth. The MDD group had a significantly lower richness index but higher Shannon and Inverse Simpson diversity indices (*P*< 0.05, Mann-Whitney U test with Bonferroni correction) (Fig. 1a). These differences remained significant after adjusting for age, BMI, sex, International Physical Activity Questionnaire (IPAQ), Mediterranean diet adherence (MDA), or smoking status (*P* < 0.05, analysis of variance [ANOVA]) (see Table S2 at https://doi.org/10.5281/zenodo.16993542). We were intrigued by the simultaneous decrease in observed species and increase in the Shannon diversity index, as these two metrics are typically positively correlated. We hypothesized that this pattern might result from the control condition being dominated by a few highly abundant species, which are depleted in patients with MDD (e.g., *F. prausnitzii*, see the following sections), resulting in a more even distribution of taxa in the MDD group (see Fig. S2 at https://doi.org/10.5281/zenodo.16993542). Mathematical modeling of the taxon distributions supports this explanation (see Fig. S3; Methods at https://doi.org/10.5281/zenodo.16993542).

Among the covariates tested for effects in alpha diversity, only the IPAQ score was significantly related to the Observed and Chao1 richness indices, with higher values at high-exercise levels than at low-exercise levels (see Fig. S4 at https://doi.org/10.5281/zenodo.16993542). However, these differences did not remain significant after adjusting for depression (see Fig. S5A and Table S2 at https://doi.org/10.5281/zenodo.16993542). None of the depression scales, including the DSM-5, BDI, MADRS, and HDRS, were significantly correlated with diversity indices in the MDD group (see Table S2 at https://doi.org/10.5281/zenodo.16993542). Interestingly, the PSS showed a negative correlation with observed and Chao1 richness. When carrying out the analysis separately for each group, only the MDD group showed the correlation (see Fig. S5B at https://doi.org/10.5281/zenodo.16993542), although it was not significant (p. adj = 0.059, R^2^ = 0.15 in both indices).

PERMANOVA analysis revealed that MDD was significantly associated with variation in microbiome composition after controlling for sex, age, BMI, IPAQ, MDA, and smoking in the same multivariate model (R² = 0.05, *P* = 0.001) (Fig. 1; see Table S3 at https://doi.org/10.5281/zenodo.16993542). However, MDD, age, and IPAQ also exhibited significant differences in group dispersion, indicating that heterogeneity of variance could influence PERMANOVA results. When tested individually, BMI, sex, and IPAQ had significant PERMANOVA *P*-values, but only sex remained significant after adjustment for depression (R² = 0.02, *P* = 0.006) (see Table S3 at https://doi.org/10.5281/zenodo.16993542). In summary, among all factors examined, MDD explained the highest proportion of the variance in microbiome composition and showed the most consistent effects on alpha diversity measures, independent of all confounders; however, in the case of beta diversity, these results should be interpreted with care due to differences in group dispersion.

### Microbiome composition associations with depression and BMI

**Fig 2:**
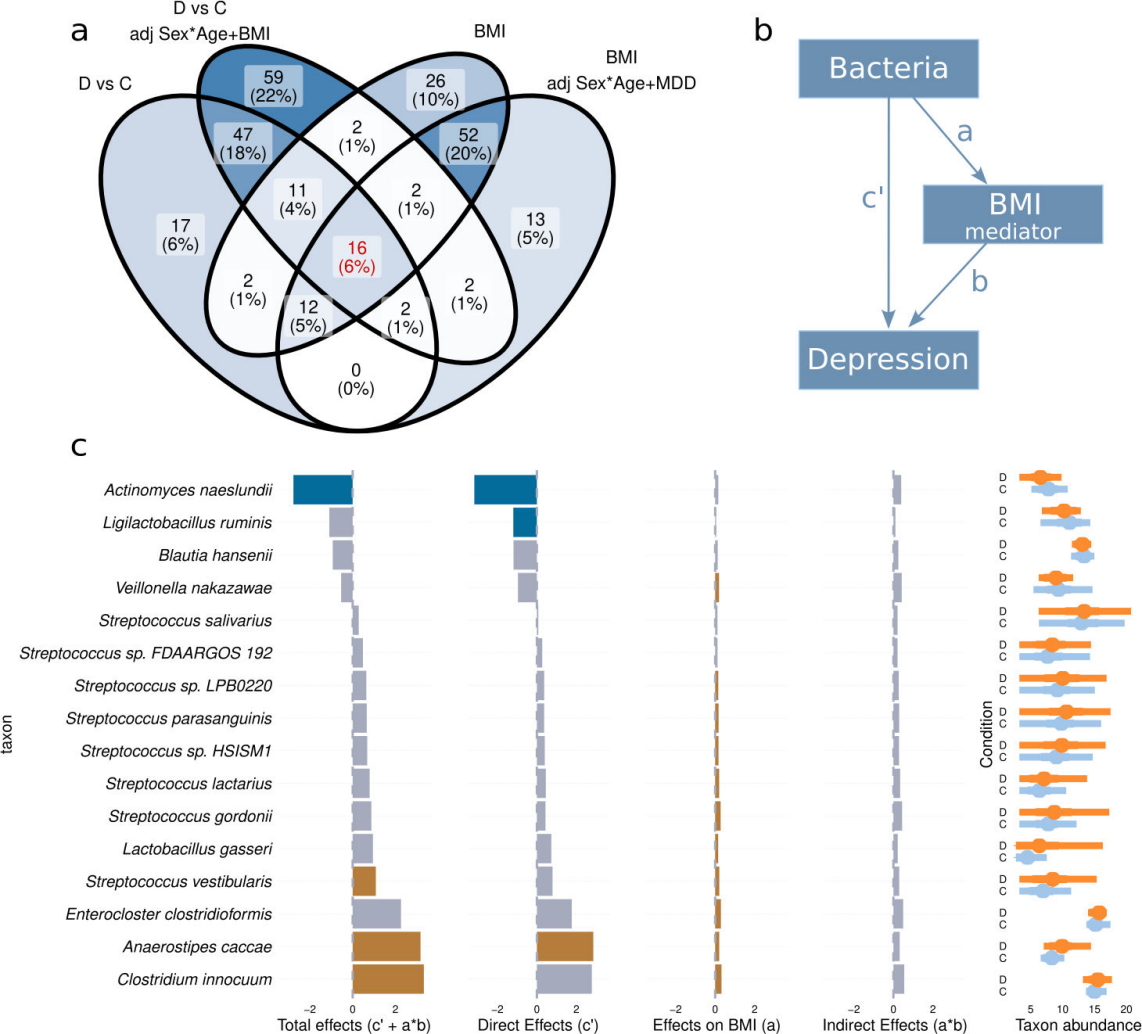
Mediation analysis. (**a**) Venn diagram comparing the differential abundance analyses performed. The 18 taxa showing differences with both MDD and BMI, before and after adjusting for the other factor, were selected for structural equation modeling-based mediation analysis. (**b**) Model used for structural equations. (**c**) Coefficients of the structural equation model. *c′* represents the direct effects of bacteria on MDD, *a* represents the direct effects of bacterial species on BMI, a*b represents the indirect effects, and c′ + a*b represents the total effect. The top-right panel represents *vst*-transformed abundances of taxa in controls and MDD patients.

We then investigated microbial species associated with MDD and BMI. Differential abundance analysis (DAA) using DESeq2 initially identified 107 species with significant differences between MDD and Controls, and 123 associated with BMI (*P* adj.< 0.05) (Fig. 2a; see Table S4; Fig. S6 and S7 at https://doi.org/10.5281/zenodo.16993542). However, in our cohort, individuals with MDD had significantly higher BMI and age (Table 1). Additionally, although sex was balanced between groups, microbiome profiles may be influenced by menopause, which typically occurs between the ages of 45 and 55 years in women.

To account for these factors, we repeated the analysis while adjusting for age, sex, and the interaction between sex and age. Under this model, 88 bacterial species were significantly associated with MDD, and 108 with BMI (see Table S4; Fig. S6 to S8 at https://doi.org/10.5281/zenodo.16993542). Finally, we performed the analysis including MDD and BMI as covariates of one another, along with age, sex, and their interaction. One hundred forty-one bacterial species were associated with MDD, and 99 with BMI (Fig. 2a; see Table S4 and Fig. S6 to S8 at https://doi.org/10.5281/zenodo.16993542). Interestingly, a few species showed significant relationships with the sex-age interaction, indicating that their abundance changes with age differently in men and women (see Fig. S9 at https://doi.org/10.5281/zenodo.16993542).

While DESeq2 tries to model count-based data with overdispersion, it does not explicitly account for the compositional nature of metagenomic data sets. To mitigate potential biases arising from relying on a single analytical framework, we also applied LinDA (linear models for differential abundance analysis), a method specifically designed to account for compositionality and library size variation in microbiome data. Overall, LinDA detected less species with differential abundance, 52 between Controls and patients with MDD after adjusting for covariates, and only six associated with BMI (see Fig. S10AB and Table S5 at https://doi.org/10.5281/zenodo.16993542). This result is coherent with recent benchmarking showing compositionally-aware methods to be more conservative and with a higher false-negative rate than DESeq2 (48). When comparing bacterial species associated with MDD with or without adjusting for covariates, around 50% of species identified by LinDA were also identified by DESeq2, whereas 20%–30% of species detected by DESeq2 were detected by LinDA (see Fig. S10C and D, S11 at https://doi.org/10.5281/zenodo.16993542). Agreement between both methods increases confidence in the identified taxa, while discrepancies highlight the influence of statistical assumptions.

### Mediation analysis shows both overlapping and orthogonal links to BMI and depression

We sought to study, in more detail, the interaction between MDD, BMI, and the microbiome. Among the 107 species associated with MDD without adjusting for any of the covariates, 41 also showed differential abundance in relation to BMI, and 16 of these remained significant for MDD and BMI when controlling for all the covariates, using the DESeq2 analysis (Fig. 2a). Thus, while some changes in the microbiome are orthogonal between depression and BMI, there is a substantial number of species that overlap, suggesting that their functions could potentially play a role in both depression and body weight gain. These overlapping species have the potential to influence the development of depression directly and also indirectly by regulating body weight. To more precisely quantify these direct and indirect effects, we performed mediation analysis with BMI as the mediator variable, MDD as the outcome, and the 16 species that showed differences for both BMI and MDD while controlling for the other factor (Fig. 2b; Materials and Methods). Structural equation analysis of the mediation model shows that out of the 16 species, 11 had significant effects on BMI, three had significant direct effects on MDD, and four had significant total effects (Fig. 2c; see Table S6 at https://doi.org/10.5281/zenodo.16993542) (adj. *P* < 0.1). Only one species, *Anaerostipes caccae*, had significant effects on both BMI and depression. Power analysis showed that the sample size was inadequate to test the indirect effects (a*b); for the effect size of most species, between 500 and 1,000 subjects would have been required to reach a power of 0.9, with alpha = 0.1 (see Fig. S12 and Table S6 at https://doi.org/10.5281/zenodo.16993542). We also performed mediation analysis with bacteria that showed differences between patients with MDD and non-depressed subjects, but not with BMI. In this case, as expected, effects on BMI were small in magnitude and mostly non-significant, whereas direct and total effects on depression were significant and very similar in magnitude (see Fig. S13 and Table S6 at https://doi.org/10.5281/zenodo.16993542). There were no overlaps between MDD and BMI associated species in LinDA analysis (see Fig. S14 at https://doi.org/10.5281/zenodo.16993542); hence, no additional mediation analysis was performed.

### Microbiome composition predicts depression status

**Fig 3:**
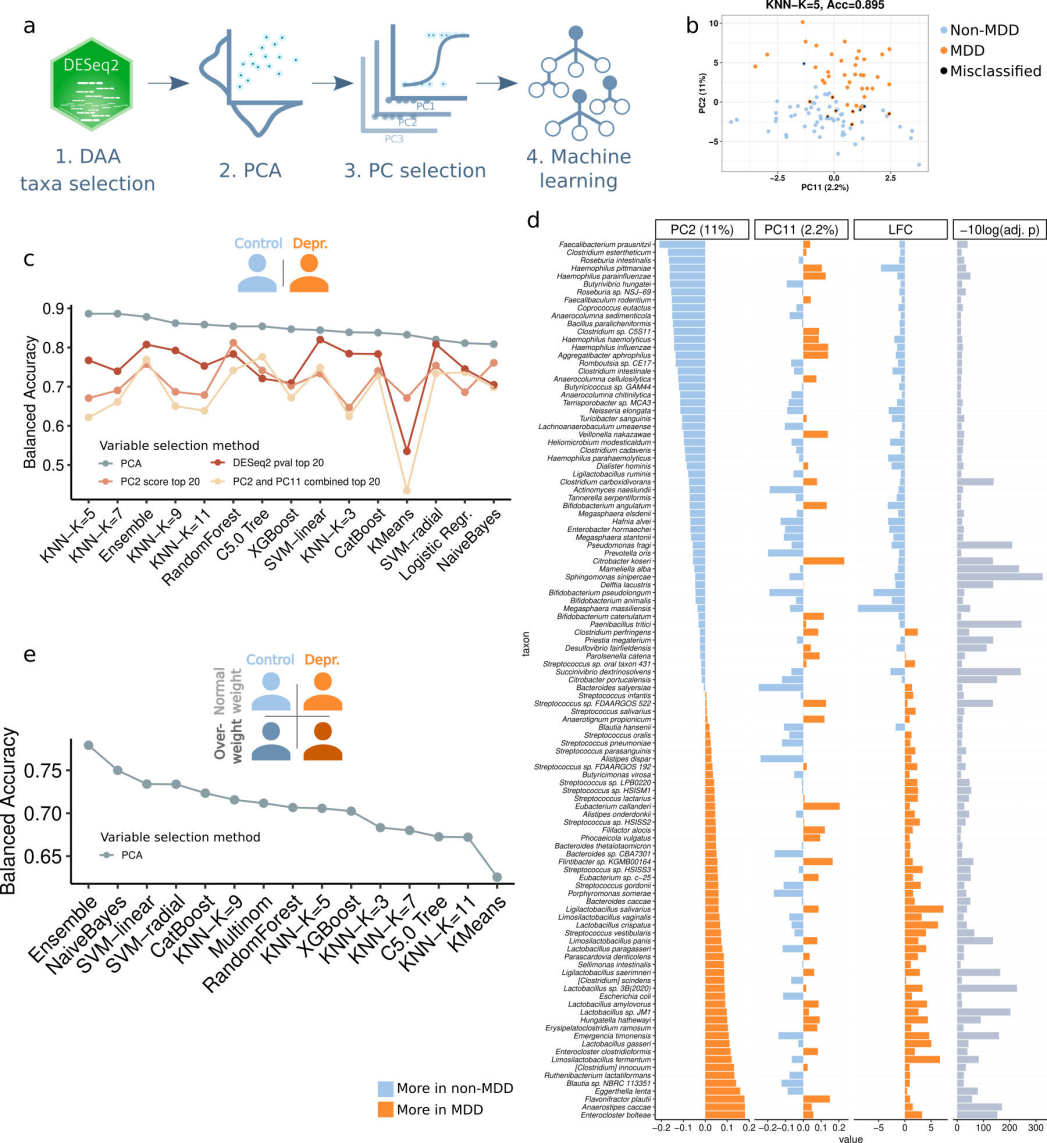
Machine learning-based prediction of MDD and overweight/obesity. (**a**) Analysis pipeline was followed to classify subjects into groups. (**b**) Results of the best performing model in the MDD vs non-depressed classification task. The two principal components used as input variables are plotted. Black dots are misclassified individuals. (**c**) Balanced accuracy of the different classifiers, for different variable selection strategies, in the MDD versus non-depressed classification task. (**d**) PCA eigenvalues for the two PCs used to classify samples. The magnitude of these values is proportional to the relevance of each bacterial species to classification. Log-fold change and *P*-values from DESeq2 are also shown. (**e**) Balanced accuracy of the different classifiers for the four-group classification task.

Given that the microbiota signatures of depression and higher BMI partially overlapped, we investigated the extent to which microbiome composition allows classification of subjects as depressed or not and overweight or normal weight based on taxonomic composition. To classify subjects according to MDD status, we followed the approach outlined in Fig. 3a. We performed a principal component analysis (PCA) with the species that were differentially abundant between both groups and used logistic regression analysis to select the principal components (PCs) showing differences between the two groups. Two PCs were selected and used to train a set of classifiers, PC2, which explained 11% of the variance, and PC11, which explained 2.2% of the variance.

Balanced accuracy ranged between 0.81 and 0.89, and AUC between 0.85 and 0.92 for all classifiers. The best-performing model was a K-Nearest Neighbors classifier with K = 5, which correctly assigned 89.5% of the subjects to their group (accuracy = 0.895, balanced accuracy = 0.889, AUC = 0.89, Cohen’s Kappa = 0.78, sensitivity = 0.83, specificity = 0.93) (Fig. 3b and c; see Fig. S15 and Table S7 at https://doi.org/10.5281/zenodo.16993542). The microbial species that most strongly discriminated between the depressed and non-depressed groups were *F. prausnitzii*, *Clostridium estertheticum*, *Roseburia intestinalis,* and *Haemophilus pittmaniae*, which were more abundant in controls than in patients and had the highest negative loadings in PC2, and *A. caccae*, *Enterocloster bolteae* (formerly known as *Clostridium bolteae*), *Eggerthella lenta,* and *Flavonifractor plautii* (formerly *Eubacterium plautii*), which were more abundant in patients and had the highest positive loadings (Fig. 3d).

Given that some species have a higher impact in classification than others, based on PC loadings, we wondered whether we could achieve a similar performance by selecting only a few bacterial species and using them as biomarkers. However, when using the 20 taxa with the lowest *P*-values in the DAA analysis, the best classifier, which was a linear kernel support vector machine (SVM), had an accuracy of 0.83 (balanced accuracy = 0.82, kappa = 0.65, AUC = 0.91), 6% lower than the best model trained with two PCs. Models trained with the 20 taxa with the highest loadings in the selected PCs also performed worse than models trained with PCs (Fig. 3c; see Fig. S15 at https://doi.org/10.5281/zenodo.16993542), and using the top 5 or top 10 taxa instead of 20 resulted in even worse performance (see Table S7 at https://doi.org/10.5281/zenodo.16993542), implying that other taxa also contain information relevant for prediction.

Although our data set was only slightly unbalanced (62 controls and 43 cases), we attempted to reduce potential bias toward the majority class using the SMOTE data augmentation technique. While some performance metrics improved for some classifiers, especially sensitivity (i.e., in this case, the model’s ability to detect the minority class), both average and best performance were slightly worse compared to models trained without using SMOTE (see Fig. S16 at https://doi.org/10.5281/zenodo.16993542).

We next divided the subjects into the following four groups: non-depressed/normal weight, non-depressed/overweight (BMI > 25), depressed/normal weight, and depressed/overweight. We used the same classification scheme as before. As input for the PCA, we selected species that were differentially abundant between depressed and non-depressed subjects, before or after adjusting for BMI, or with respect to BMI, before or after adjusting for depression. We selected the 11 PCs that were significantly correlated with any of the four groups and trained the same array of models as before (see Table S7 at https://doi.org/10.5281/zenodo.16993542). The best-performing model was the ensemble, followed by Naive Bayes, with a balanced accuracy of 0.78 and 0.75, a Kappa of 0.58 and 0.52, and an AUC of 0.84 and 0.8, respectively (Fig. 3e; see Fig. S17 and Table S7 at https://doi.org/10.5281/zenodo.16993542). Using SMOTE data augmentation resulted in similar performance (see Fig. S17 at https://doi.org/10.5281/zenodo.16993542).

Finally, we repeated all the workflow selecting bacterial species to include in the PCA using LinDA, instead of DESeq2. Performance metrics decreased slightly, with a maximum balanced accuracy of 0.87 (Kappa = 0.74, AUC = 0.89) in the two-group classification problem and of 0.76 in the 4-group problem (Kappa = 0.53, AUC = 0.85) (see Fig. S18 and S19 at https://doi.org/10.5281/zenodo.16993542).

### Functional analysis of metagenomes

**Fig 4:**
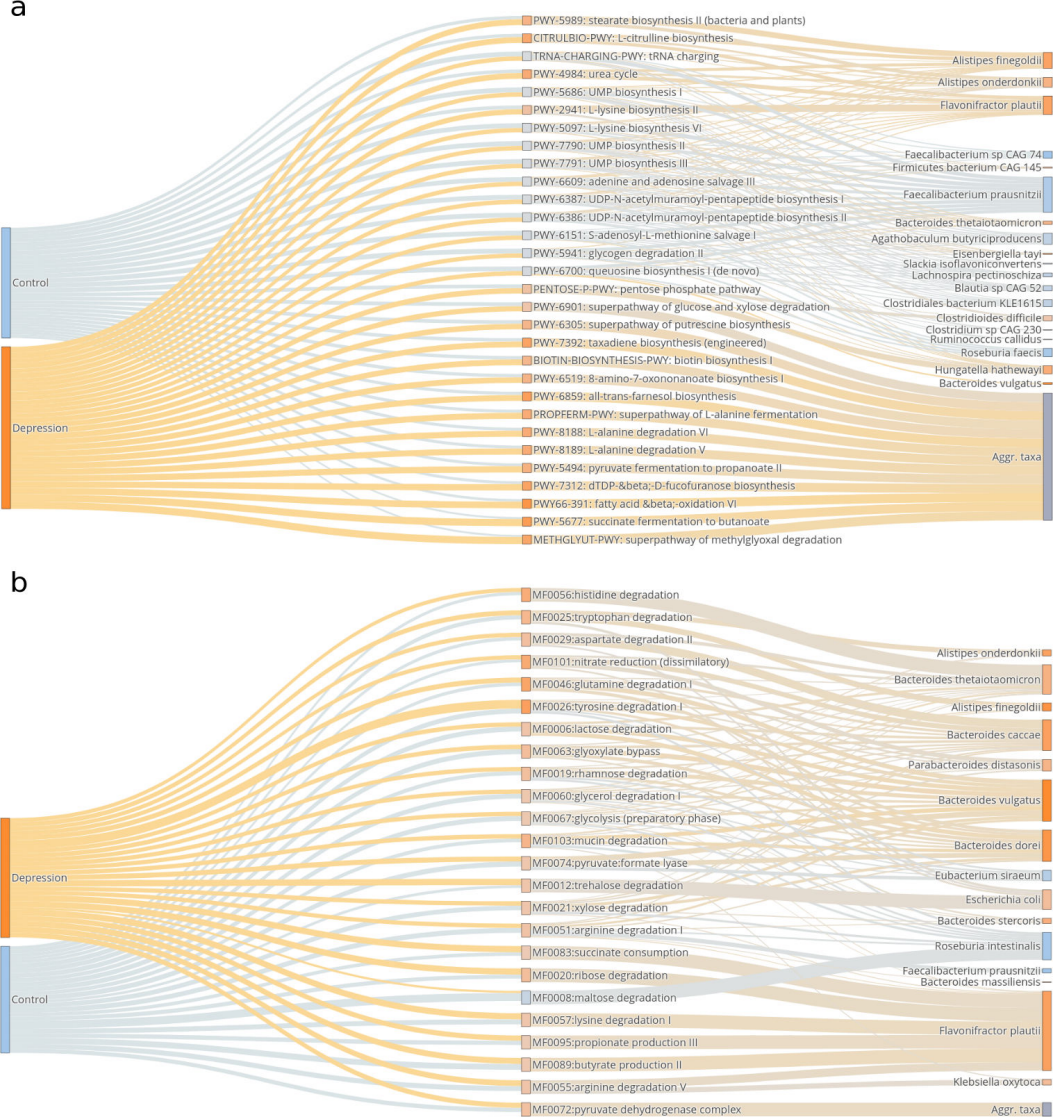
Functional annotation. Top 30 significant (*P* < 0.05) MetaCyc (**a**) and GOmixer (**b**) terms, after adjusting for BMI, sex, age, and the sex*age interaction, are shown. Line widths are proportional to the contribution of each group of subjects to each pathway (left) and to the contribution of each bacterial species to each pathway (right). Orange is for pathways and species that are more abundant in patients with MDD, whereas blue is for pathways and species that are more abundant in non-depressed controls.

To identify which biological functions and pathways might be mechanistically related to depression, we annotated the metagenomes using HUMAnN3 (62) and performed DAA controlling for BMI, sex, and age with interaction. By controlling for these covariates, we identify differences between the control and the MDD group that are not influenced by BMI, age, or sex. The MetaCyc pathways that were more abundant in the MDD group were succinate fermentation to butanoate (PWY-5677), L-citrulline biosynthesis (CITRULBIO-PWY), fatty acid & β-oxidation (PWY66-391), the urea cycle (PWY-4984), all-trans-farnesol biosynthesis (PWY-6859), taxadiene biosynthesis (PWY-7392), superpathway of putrescine biosynthesis (PWY-6305), and methylglyoxal degradation (METHGLYUT-PWY) (Fig. 4a; see Table S8 at https://doi.org/10.5281/zenodo.16993542).

The MetaCyc pathways that were more abundant in the non-depressed group were L-lysine biosynthesis (PWY-5097), S-adenosyl-L-methionine salvage I (PWY-6151), adenine and adenosine salvage III (PWY-6609), UMP biosynthesis (PWY-7791, PWY-7790, and PWY-5686), queuosine biosynthesis (PWY-6700), glycogen degradation (PWY-5941), purine ribonucleosides degradation (PWY0-1296), D-fructuronate degradation (PWY-7242), and thiamine phosphate formation from pyrithiamine and oxythiamine (PWY-7357), among others (Fig. 4a; see Table S8 at https://doi.org/10.5281/zenodo.16993542).

We also evaluated the differential abundance of pathways in gut-specific metabolic modules using GOmixer (63). Patients with MDD showed a higher abundance of histidine, tryptophan, aspartate, and glutamine degradation pathways (MF0056, MF0025, MF0029, and MF0046), as well as nitrate reduction (MF0101) and mucin degradation (MF0103), among others, while maltose degradation (MF0008) was more abundant in controls (Fig. 4b).

When looking at species contributing to differentially abundant pathways, we observed that some species had a proportionally high contribution to many pathways. For instance, *F. prausnitzii* and *R. intestinalis* contributed to most of the pathways that were depleted in patients with MDD, whereas other species, such as *F. plautii*, *Alistipes onderdonkii, Hungatella hathewayi*, and members of the genus *Bacteroides*, contributed the most to functions positively associated with depression (Fig. 4a and b). Despite the taxonomic profiling and the functional analysis being conducted with different software and databases, these taxa are largely coincident with those found to be differentially abundant between controls and MDD patients (see Fig. S7 and Table S4 at https://doi.org/10.5281/zenodo.16993542).

We also looked at pathways associated with BMI, but after adjustment by depression, age, and sex with interaction, there were no significant MetaCyc or GOMixer terms (see Table S8 at https://doi.org/10.5281/zenodo.16993542).

## DISCUSSION

This study identifies distinctive and shared microbiome signatures of depression and overweight/obesity and identifies the functional microbiome pathways underlying the disease status, contributing to explain these bidirectional associations and establishing the potential of the microbiome for patient stratification.

Previous epidemiological studies have demonstrated differences in microbiome composition between depressed patients and healthy individuals; however, the majority of studies examined only taxonomic features and the role of co-existing comorbidities, such as obesity, was largely unexplored (18, 64, 65). Here, we show that depressed patients have lower microbial richness but higher diversity, independently of age, sex, BMI, and lifestyle variables. Higher diversity is generally considered positive (66), although it has also been associated with microbiome instability (67). Most previous studies and systematic reviews have not found differences in richness and diversity in depressed subjects, but the findings are mostly inconsistent (19, 21, 24, 25, 28, 29, 36). In a systematic review, for instance, only 2 of 12 studies reported differences in Shannon index—one reported an increase and the other a decrease—while four reported a decrease in richness (68). The only study using shotgun metagenomics, however, reported a trend in alpha diversity similar to our study, a decrease in richness, and a non-significant increase in diversity (69).

Importantly, our study shows that BMI is a key confounding factor in depression studies and that may partially mediate microbiome associations with depression. It is therefore possible that many microbiome-depression associations are a consequence of this BMI bias and do not reflect direct associations with depression. By appropriately adjusting for BMI, age, and sex, we were able to identify depression-specific taxonomic signatures. For example, *E. lenta* has been positively associated with depression in several studies (18, 21, 23, 25, 28, 36, 68, 70), as commented in the introduction. Although we replicated this finding, the difference between depressed and non-depressed subjects faded after controlling for BMI. Other bacterial species that have been positively associated with depression in previous studies are confirmed to be so independently of BMI, including *F. plautii* and *H. hathewayi* (18, 26, 35, 71–73). Many of the species that were significantly depleted in depression after controlling for BMI were short-chain fatty acid producers. Some of them have been associated with depression in previous studies, including *F. prausnitzii*, *R. intestinalis*, *Hafnia alvei,* or the genus *Romboutsia* (18, 26, 68, 70, 74, 75), and some have been newly discovered in our study, such as species of the genus *Anaerocolumna* and *Butyrivibrio hungatei*. Among these, *F. prausnitzii* is one of the most studied in relation to mental health (76). *F. prausnitzii* strains have been shown to exert preventive and therapeutic effects on chronic unpredictable mild stress-induced depressive and anxiety-like behavior in rodents (77), although evidence from human studies is still lacking. Interestingly, one study identified a cluster of single-nucleotide polymorphisms (SNPs) in *F. prausnitzii* associated with BMI (78). These SNPs were located in genomic regions enriched in energy production and conversion function, possibly affecting the metabolic efficiency of the bacteria and, consequently, increasing the host’s capacity to absorb energy from the diet. *F. prausnitzii* has also been reported to decrease with age in postmenopausal women with HIV (79). In our cohort, however, this species did not show a significant relationship with age in either male or female participants (see Table S4 at https://doi.org/10.5281/zenodo.16993542).

At least 16 species were directly associated with both higher BMI and depression. Mediation analysis allowed us to quantify and test the distinct effects of these bacteria on both phenotypes. Half of these species belonged to the genus *Streptococcus*. Although *Streptococcus* species have been linked to both depression and neurotransmitter regulation, as well as obesity (80, 81), in most cases, the coefficients of the structural equation model were significant only for BMI. Importantly, effects on BMI and depression were both significant for *Anaerostipes caccae*, showing that this species can affect depressive disorders both directly and indirectly through BMI. *A. caccae* has been associated with binge eating disorders and is a predictor of depression (18, 82), but it is also considered an important producer of short-chain fatty acids and has been positively linked with physical activity (83, 84).

The substantial differences in microbial abundances between MDD and non-depressed subjects allowed us to successfully classify individuals in both groups based only on their microbiome composition with high accuracy. Indeed, 94 of the 105 subjects were correctly classified, representing a success rate of ~90%. Taxa contributing the most to the classification of depressed subjects include *A. caccae, E. bolteae, E. lenta,* and *F. plautii,* which belong to genera associated with depression (18, 35, 70). Among the bacterial species associated with a healthy state, *F. prausnitzii* contributed the most to classification.

However, replacing principal components with the abundances of individual taxa reduced classification accuracy to 0.83 (balanced accuracy = 0.82). This result could be partly explained by the fact that using a lower number of variables simplifies the learning task and reduces overfitting. Alternatively, it needs to be considered that PCs contain information from a much broader set of taxa, capturing ecosystem-wide properties such as functional redundancies and complex microbial interactions. This suggests that approaches using the whole microbiome structure may be more promising than those that rely on specific microbial species as potential future diagnostic tools, despite being more difficult to interpret or to implement.

Classification of individuals into MDD/non-depressed and normal weight/overweight resulted in a balanced accuracy of 0.78, indicating that the four groups have distinct microbiome profiles. The decrease in performance, compared to the two-group classification task, reflects the increased complexity of the task, as well as the smaller sample size within each group.

In addition to the taxonomic analysis, we explored the possible mechanisms underlying the associations of the microbiome with MDD using functional annotation and pathway abundance analysis. Data shows that few taxa contribute proportionally the most to differentially abundant pathways: most pathways associated with a healthy mental status were performed by *F. prausnitzii*, and most functions associated with depression were mainly performed by a few species, including *F. plautii, A. onderdonkii, H. hathewayi*, *R. intestinalis,* and members of the *Bacteroides* genus (Fig. 4). Interestingly, some of these taxa are the ones contributing the most to classification into controls and patients with MDD (Fig. 2).

Many of the MetaCyc and GOmixer pathways that showed differences between patients with MDD and non-depressed subjects were related to nucleotide or amino acid metabolism, which have a direct impact on neurotransmitter synthesis. Among MetaCyc terms, the higher queuosine biosynthesis in non-depressed subjects is of particular interest, as this function is performed exclusively by bacteria. Queuosine is used to synthesize queuine, which is incorporated into tRNAs in the wobble position to promote accurate mRNA translation. Its deficiency results in the depletion of tetrabiohydropterine (BH4), a necessary cofactor in the synthesis of several neurotransmitters, including dopamine and serotonin, which are thought to be at the core of depression pathology (85). Queuosine depletion is expected to result in symptoms similar to psychiatric diseases, and queuine has been shown to be protective against neurodegeneration *in vitro* (86). In our samples, queuosine synthesis is performed by both MDD-depleted and MDD-increased bacteria. *F. prausnitzii*, *Agathobaculum butyriciproducens*, *Blautia sp. CAG 52*, and *Roseburia faecis* are among the depleted ones, whereas *H. hathewayi* and *Bacteroides vulgatus* are among the MDD-increased. Overall, the balance is negative, with lower synthesis in patients with MDD vs controls (Fig. 2b).

L-lysine, another important pathway depleted in patients with MDD, is an essential amino acid, and its dietary deficiency has been shown to increase stress response in rats (87), and reduce stress and anxiety in healthy human subjects (88), although results are conflicting (89). Like in the queuosine biosynthesis pathway, abundance changes are the net result of changes in the abundances of both MDD-increased and MDD-depleted bacteria, including *F. prausnitzii*, *Blautia sp. CAG 52*, *A. butyriciproducens*, *H. hathewayi, Alistipes species*, *F. plautii*, and others (Fig. 2b).

On the other hand, glutamine, tryptophan, and histidine degradation were among the most over-represented GOmixer terms in patients with MDD, supporting previous findings. For example, glutamine depletion has been reported in patients with depression (90, 91), and glutamine supplementation has antidepressant effects in preclinical studies (92). Glutamine is involved in the production of glutamate and GABA, two of the most important neurotransmitters. Tryptophan is required for the synthesis of serotonin and kynurenine, both important neuroactive compounds (93). Clinical trials have shown that diets high in tryptophan result in fewer depressive symptoms and reduced anxiety (94). Similarly, daily ingestion of histidine for 2 weeks is also known to improve mood (95). Accordingly, increased breakdown of these amino acids by the gut microbiome may be an important mechanism underlying their association with depression. In our data set, the increased abundance of *Alistipes onderdonkii* and *A. finegoldii* in patients with MDD accounted for most of the increase in tryptophan degradation, whereas species from the genus *Bacteroides*, mainly *B. caccae*, *B. thetaiotaomicron*, *B. dorei,* and *B. vulgatus*, seem to be responsible for histamine and glutamine degradation.

We also provide the first evidence of increased mucin degradation potential in the microbiome of depressed patients. Mucins are highly glycosylated proteins that form the mucus layer in the intestine, a critical barrier between the microbiome and host cells, and essential for the maintenance of the intestinal epithelium. Disruption of this layer can lead to leaky gut and to intestinal dysbiosis, with consequent imbalances in microbial metabolites that affect immunity and mental health, including SCFAs and tryptophan (96). Stress, a major risk factor for depression, can also shift the O-glycosylation patterns of mucins in rats (97), which can also influence mucus degradation. According to our functional data set, *Klebsiella oxytoca*, together with *B. vulgatus* and other *Bacteroides* species, is responsible for the increase in mucin degradation potential.

Intriguingly, Fig. 4b shows how certain short-chain fatty acid synthesis pathways, dependent on *F. plautii*, are more abundant in depressed subjects. Although *F. plautii* is known to produce butyrate and propionate (98), it has been consistently associated with depression (19, 21, 22, 25, 26, 28). In an Indian cohort, it was also linked to colorectal cancer (99), a relationship the authors attributed to its ability to degrade anticarcinogenic flavonoids. Given that flavonoids have demonstrated protective effects against depression (100, 101), their excessive degradation may also contribute to the association between *F. plautii* and depression. Additionally, *F. plautii* has been reported to be increased in type 2 diabetes (102) and reduced in obese subjects compared to normal weight controls (103). Further studies are needed to clarify the complex and context-dependent roles of *F. plautii* in the gut microbiome.

In conclusion, our study reveals unique microbiome signatures predictive of depression and functional pathways that affect mental health and potentially account for the disease pathogenesis. *F. prausnitzii* was consistently associated with a healthy mental state in our study, in accordance with previous human and preclinical studies (19, 22). *F. prausnitzii* performs important functions, including roles in neurotransmitter metabolism, warranting further investigation for possible bench-to-bedside translational studies to alleviate depression. Moreover, our study identifies microbiome signatures associated with both depression and higher BMI, suggesting that microbiome profiling could help classify different BMI-stratified depression phenotypes and refine diagnosis and therapeutics.

### Limitations of the study

Most patients were under pharmacological treatment for depression, and only a few were not exposed to drugs; therefore, the possible contribution of this factor to gut microbiome variations in the patients could not be assessed appropriately. The sample size was limited, especially when variations in BMI within the MDD and control groups were considered, and therefore, we lacked power in some of the analyses, as commented in the results section. Finally, the cross-sectional design of the study limits our ability to infer directionality in the relationships observed between MDD, BMI, and the microbiome. Longitudinal studies and interventions directed to modulate specific microbial features are needed to confirm these associations. Despite these limitations, our study provides valuable insights into the three-way relationship between the gut microbiome, depression, and high BMI.

## MATERIALS AND METHODS

### Participant recruitment

We recruited 119 participants from September 2017 to October 2020. Sequencing quality was low for 14 of them and were excluded. Among the 105 remaining, 43 were diagnosed with MDD, and 62 volunteers were non-depressed controls. Patients with MDD and non-depressed controls were recruited in different clinical centers (Universitat de les Illes Balears, Hospital La Princesa, Hospital Clinic Barcelona, University Clinic of Navarra, and University of Oviedo).

### Inclusion criteria

The study included individuals aged 18–65 years. The case group comprised patients diagnosed with MDD, either first episode or recurrent, of varying severity. Diagnosis was confirmed using the Structured Clinical Interview for DSM-5 (SCID-5) administered by trained clinicians at the hospital of origin. Participants with or without metabolic comorbidities, including obesity, type 2 diabetes mellitus, dyslipidemia, or metabolic syndrome, were eligible. Participants taking medication for depression were also eligible.

The control group was composed of individuals from the same age range, with no current or past psychiatric disorders. Controls were recruited to match the MDD group by sex distribution, and recruitment was stratified to minimize differences in age and education level.

### Exclusion criteria

Exclusion criteria included functional gastrointestinal disorders or other significant gastrointestinal complaints, past or present major psychiatric disorders like schizophrenia, bipolar disorder, obsessive-compulsive disorder, post-traumatic stress disorder, or generalized anxiety disorder, type I diabetes or other primary endocrine pathologies, leukocytosis (>10,000), fever (>38° in the interview), or vaccination in the 4-week period preceding sample collection. Vaccinations, particularly those involving live-attenuated viruses or adjuvanted formulations, can transiently alter immune function, gut microbiota composition, and neuroimmune interactions (104, 105). Additionally, participants who had received antibiotic treatment in the 3 months before recruitment or who had drastically changed their dietary habits in the month previous to recruitment were also excluded.

### Assessment of clinical history, disease status, and severity

We obtained information on the BDI to calculate a self-score for the degree of depression over the previous 2 weeks. The severity of depression was also assessed using the 17-item HDRS and the clinician-rated 10-item MADRS. To get the HDRS-17 score, each item on the validated questionnaire was scored on a three- or five-point scale, depending on the item. We used the calculated HDRS-17 score as a continuous variable and also as a qualitative variable, classifying depressed subjects into different categories. To get the MADRS index, we scored each item on the validated scale from 0 to 6, thus ranging the overall score from 0 to 60. Stress was assessed using the validated PSS, and subjects were classified as low stressed (PSS scores ranging from 0 to 13), moderately stressed (scores from 14 to 26), and highly stressed (scores from 27 to 40).

Furthermore, we collected sociodemographic (age and sex) data along with BMI and the presence of the primary metabolic comorbidities associated with depression, such as obesity, type 2 diabetes, and metabolic syndrome, as well as conditions like elevated blood pressure, elevated blood sugar, and abnormal cholesterol or triglyceride levels. We also recorded the use of medication and categorized medications and drugs according to their purposes, modes of action, and conditions they treat. The Bristol stool scale was also applied to record stool consistency, and accordingly, participants were divided into three categories (constipation, diarrhea, and normal intestinal transit).

### Lifestyle and diet data collection

We recorded information on several modifiable lifestyle factors related to depression, such as smoking, alcohol abuse, diet, and physical activity. Regarding diet, we evaluated the adherence to the Mediterranean diet using the validated 14-point MEDAS. There are two questions about food intake behaviors said to be typical of the Spanish Mediterranean diet and 12 questions about the frequency of food consumption that constituted the MEDAS. This has been used previously in the PREDIMED study as an effective tool for the quick estimation of adherence to the Mediterranean diet. A MEDAS-derived PREDIMED score, which ranges from 0 to 14, was determined by replying to the 14 questions/items of the MEDAS questionnaire. Finally, we used the calculated MEDAS-derived PREDIMED score as a continuous variable and also to classify subjects into the following categories: subjects with low adherence to MedDiet or “very low diet quality” (scores ≤3), subjects with Moderate compliance to MedDiet or “average MedDiet adherence” (score range 4-7), and subjects with “optimal adherence to MedDiet” (scores ≥8). The physical activity was assessed using the 7-item IPAQ, based on open-ended questions surrounding individuals’ last 7-day recall.

### Shotgun sequencing

Stool samples were self-collected at home using sterile containers and immediately frozen until transport to the hospital. At the hospitals, samples were stored at −80°C and shipped in dry ice to the Institute of Agrochemistry and Food Technology in Paterna (IATA-CSIC), where they were stored at −80°C until processing. DNA extraction was performed using the Magpure Stool DNA LQ kit, and the NGS library was prepared using the MGIEasy Universal DNA Library Prep Set. Shotgun metagenomics sequencing was performed in MGI’s DNBSEQ-G50 sequencing platform to generate paired-end short reads of a maximum of 150 bp and targeting a minimum sequencing depth of 20M reads, which is above the minimum recommended for stable taxonomic and functional profiling (106, 107), at the MGI Tech Co., Ltd. manufacturing facility in Riga (Latvia).

### Taxonomic and differential abundance analyses

Trimmomatic (v0.39) (108) was used to trim paired-end reads, using a sliding window of 20 bp and a quality threshold of 30. Reads shorter than 75 bp were discarded. FastQC (v0.12.1) (109) and MultiQC (v1.25.1) (110) were used to assess sequencing quality before and after trimming. Surviving reads were mapped against the GRCh38 human genome assembly using bowtie2 (v2.5.1) (111), and mapped reads were discarded. The unmapped reads were then classified at the level of species using Kraken2 (v2.1.3) (112), with confidence set to 0.1, and Bracken v2.8 (113), using an updated version of the standard Kraken database (https://genome-idx.s3.amazonaws.com/kraken/k2_standard_20220607.tar.gz). Taxa with a prevalence lower than 0.05 were filtered out before any downstream analyses; therefore, low-prevalence taxa have been removed from all the results presented throughout the paper. Taxon saturation was assessed using rarefaction curves generated with the *rarecurve* function from the vegan package (v2.6-8), using a step size of 10.000.

The filtered count matrix was rarefied to the minimum depth, which was 8,999.833 after prevalence filtering, and alpha and beta diversity were calculated with the *phyloseq* package (v.46.0) (114). To compare alpha diversity indices between groups, a Wilcoxon test with Bonferroni correction was used. In order to control for the effects of covariates, a series of linear models were fitted with the alpha diversity indices as response variables and depression, age, BMI, sex, exercise levels (IPAQ), Mediterranean diet adherence, and/or smoking status as independent variables. The residuals of models using only depression were compared against the residuals of more complex models using ANOVA. Additionally, models using IPAQ or PSS scales were separately fit for controls and depressed subjects. Samples with missing data in any of these variables were removed when adjusting for that variable.

For beta diversity analysis, we computed Bray-Curtis dissimilarities. To assess the assumption of multivariate homogeneity of variances, we used the *betadisper* function from the *vegan* R package (v2.6-8), testing each of the variables in our data set as a grouping factor. Next, we performed PERMANOVA analyses using the *adonis2* function from *vegan* (115), including the same covariates used in the alpha diversity analysis. We tested both individual variables and combinations of covariates by specifying formulas such as “Bray.dist ~ Depression + Sex + Age,” which were passed to *adonis2*.

DESeq2 (v1.42.1) (116) was used for differential abundance analysis (DAA) of the identified taxa, starting from the raw (non-rarefied) abundance matrix filtered by taxon prevalence. Raw counts were used as input because DESeq2 internally normalizes for sequencing depth by estimating size factors using the median-of-ratios method. DESeq2 was run multiple times, using depression or BMI as the only independent variables, adding age, sex, and interaction between age and sex as covariates, or these covariates plus BMI and MDD together. *P*-values were adjusted using the Benjamini-Hochberg (BH) method. The “normal” method (116) was used to moderate Log-fold change (LFC) estimates. As a complementary approach, DAA was also conducted with LinDA (v0.2.0) (117), using the same raw count matrix as input. LinDA internally applies a centered log-ratio (CLR) transformation after adding a small pseudocount; hence, no prior normalization is needed. We used a winsorization cutoff of 0.97 to reduce the influence of outliers and also included BMI and sex*age as covariates. The results of DESeq2 and LinDA were compared using Venn diagrams and bar plots.

### Mathematical modeling of taxon distributions

The microbiome community is generally composed of a few dominant species and many species at lower abundances (118). This distribution can be modeled as a negative exponential curve of the general form:


(1) $$abundance=e^{-{\left(ax\right)}^{b}}$$

where *x* = {1, 2, 3, …} is the rank of each species (i.e., the most abundant one, the second most abundant one, etc.) and *a* and *b* are parameters that control the shape of the distribution. First, species abundances were normalized to 1 by dividing by the highest abundance in each corresponding sample. We then used the least squares method to estimate the value of the parameter *a* for each individual sample, while keeping *b* fixed at 0.5, a value determined empirically through trial and error. Parameter estimation was performed using the *nls* R function (in the *stats* package; v4.3.3) with the initial value for *a* set to 0.1. In all cases, the algorithm converged within 4 to 11 iterations, reaching a convergence tolerance below 1e-5. The residual standard error ranged from 0.02 to 0.05 (mean±SD: 0.03±0.005) on a scale of 0 to 1, as reported by the *nls* summary output. Next, equation 1 was used to simulate distributions with feasible numbers of observed species (from 1 to 553, 10 more than the maximum number of species observed in our data set), and with realistic values for *a* (from 0 to 0.87, 20% higher than the maximum value of *a* calculated for any of our samples). Finally, the Shannon diversity index was calculated for each of the simulated distributions and plotted against the number of species and *a* values (see Fig. S3B at https://doi.org/10.5281/zenodo.16993542).

### Mediation analysis

Taxa were selected based on DESeq2 adjusted *P*-values from differential abundance analyses across relevant comparisons (Fig. 2a; see Fig. S13A at https://doi.org/10.5281/zenodo.16993542). Mediation analysis was then performed to evaluate whether the association between specific taxa and depression was mediated by BMI. The mediation model was defined as follows:


(2) $$\begin{array}{lcl} \text{Depression} & = & c^{\prime }*\text{taxon abundance}+b*\text{BMI}\\ \text{BMI} & = & a*\text{taxon abundance} \end{array}$$

Where *c′* represents the direct effect of each species on depression, *a* is the direct effect of each species on BMI (the mediator), and *b* is the effect of BMI on depression. The indirect effect of each taxon on depression is given by a*b, and the total effect is c′+a*b (Fig. 2b). Structural equation modeling was performed using the *lavaan* R package (v0.6-19) (119), specifically the *lavaan::sem*() function. Estimates of the path coefficients, standard errors, z-values, and associated *P*-values were extracted from the model summary, and multiple testing correction was applied to *P*-values using the Benjamini-Hochberg method.

Power analysis was conducted by simulating data sets with 100 to 1,000 individuals, with 100 replicates per sample size. For each simulation, bacterial abundances were generated by bootstrapping from the original variance-stabilized (vst) abundances. Mediator values were then generated using the lower part of equation 2, with the previously estimated *a*, the simulated bacterial abundances, and adding a residual error term bootstrapped from the *lavaan* model residuals. Next, the outcome variable Ŷ was simulated using the upper part of equation 2:


(3) $$Ŷ=c^{\prime }*simulated\;abundance+b*simulated\;BMI$$

The probability of depression was calculated by applying the logistic function to Ŷ:


(4) $$\text{Prob}\left(Y=1\right)=\frac{1}{1+e{\;}^{-\hat{Y}}}$$

Finally, the binary outcomes (absence/presence of MDD) were generated using the *rbinom*() function in R, with probabilities from equation 4. *Lavaan* SEM modeling was again applied to the simulated data set, and for each taxon-term pair, the proportion of simulations yielding a statistically significant *P*-value (*P* < 0.05) was computed for each population size. The minimal required sample size was defined as the smallest population size at which at least 90% of simulations produced a significant *P*-value for the term of interest.

### Predictive analysis

To classify samples into two groups (Depression/Control) or four groups (Depression/Control and BMI <25/BMI >25), we first selected taxa that were differentially abundant between groups, at an adjusted *P*-value < 0.05. In the case of the two-group classification using LinDA analysis, taxa were filtered using an adjusted *P*-value < 0.01, as this slightly improved performance. PCA was then performed on the DESeq2 variance-stabilized abundances (vst), using the *prcomp* R function. For binary classification, a set of logistic regression models was fitted using each principal component as a predictor, and components whose coefficients were significant (*P* < 0.05) were retained. For the four-group classification, significant PCs were selected using a multinomial log-linear model from the *nnet* R package. In this case, PCs with *P*-values < 0.1 were retained, as this led to slightly better performance compared to the 0.05 cutoff.

Several machine learning models were then trained using the selected PCs. Given the relatively low number of samples, we used the leave-one-out method to maximize training data; therefore, each model was trained N times, and each time N-1 samples were used for training, and one sample was used for the test. All the metrics reported in figures and tables result from this leave-one-out procedure. Table 2 shows the package versions and parameters used for each classifier. For CatBoost and XGBoost, grid search was performed to find the best combination of parameters independently for two-group and four-group classification tasks. To compute the ensemble classification, each model’s output class was weighted by its performance, as measured by balanced accuracy, and the class with the highest combined score was selected.

**TABLE 2:** Parameters used in machine learning models

Model	Package/version	Two-group parameters	Four-group parameters
KNN	class 7.3–22	K = [3, 5, 7, 9]	K = [3, 5, 7, 9]
K-Means	stats 4.3.3	centers = 2 iter.max = 100 nstart = 10	centers = 4 iter.max = 100 nstart = 10
NaiveBayes	e1071 1.7-16	laplace = 0	laplace = 0
C5.0 Tree	C50 0.1.8	trials = 20	trials = 20
RandomForest	randomForest 4.7-1.2	ntree = 500 mtry = 1 nodesize = 1	ntree = 500 mtry = 4 nodesize = 5
XGBoost	xgboost 3.0.2.1	learning_rate = 0.3 max_depth = 2 nrounds = 50 min_child_weight = 1 subsample = 0.6 colsample_bytree = 1 reg_lambda = 3 reg_alpha = 0 objective = "binary:logistic" scale_pos_weight: if SMOTE not used	learning_rate = 0.3 max_depth = 2 nrounds = 30 min_child_weight = 1 subsample = 1 colsample_bytree = 0.6 reg_lambda = 1 reg_alpha = 0 objective = "multi:softprob" scale_pos_weight: if SMOTE not used
CatBoost	catboost 1.2.8	iterations = 100 learning_rate = 0.05 depth = 2 loss_function = "Logloss" eval_metric="AUC" random_seed = 123 use_best_model=TRUE od_type = "Iter" od_wait = 20 bootstrap_type = "Bayesian" l2_leaf_reg=3 grow_policy = "Depthwise" auto_class_weights = "Balanced"	iterations = 100, learning_rate = 0.05, depth = 4, loss_function = "MultiClass," eval_metric = "MultiClass," random_seed = 123, use_best_model = TRUE, od_type = "Iter," od_wait = 20, bootstrap_type = "Bernoulli," subsample = 0.6, l2_leaf_reg = 3, grow_policy = "Depthwise," auto_class_weights = "Balanced"
SVM-linear	e1071 1.7–16	kernel = ”linear” weights (if not using SMOTE)	kernel = ”linear” weights (if not using SMOTE)
SVM-radial	e1071 1.7–16	kernel = ”radial” weights (if not using SMOTE)	kernel = ”radial” weights (if not using SMOTE)
Logistic Regr.	stats 4.3.3 glm()	family = binomial	Not applicable
Multinom.	nnet 7.3–19 multinom()	Not applicable	default

To avoid any bias due to class imbalance, three different methods were used: (i) imbalance-aware evaluation metrics were used, such as balanced accuracy and Cohen’s Kappa, to provide a more reliable assessment of model performance across classes; (ii) for models that support sample weighting (Random Forest, XGBoost, CatBoost, and SVMs), Class weights were assigned inversely proportional to class frequencies; and (iii) all analyses were repeated using the SMOTE technique to generate synthetic samples of the minority classes. Specifically, the *SmoteClassif*() function of the *UBL* package (v0.0.9) was used, with parameters *K = 5* and *dup_size = “balance.”*

The *caret* R package (v7.0-1) was used to compute most performance metrics (120), except for the Area Under the Curve (AUC), which was computed using the *roc*() function (for binary classification) or the *multiclass.roc*() function (for four-group classification) of the *pROC* R package (v1.18.5). Since K-Means does not natively provide class probabilities, which are required to compute AUC, an approximate probability was derived by dividing the distance from a sample to the cluster centroids by the sum of all centroid distances, and subtracting the result from 1. For the ensemble approach, class probabilities were estimated by dividing the score (i.e., weighted sum of all model outputs) of each class by the sum of all scores.

Additionally, the same models were trained with the 5, 10, or 20 top taxon abundances, instead of principal components. These top taxa were either the ones with the lowest *P*-values in the DESeq2 analysis or the ones with the highest absolute scores in the selected principal components.

### Functional analysis

For functional annotation of metagenomes, the HUMAnN3 pipeline was used (62), with default options, starting from the trimmed and human-depleted reads. Then, the *humann_regroup_table* command was used to translate MetaCyc terms into MetaCyc reactions, Gene Ontology, KEGG Orthology (KO), and CAZy terms, and the *humann_renorm_table* command was used to compute copies per million (CPM). The *omixerRpm* R package was used to transform KO terms into gut-specific metabolic (GOmixer) modules (63, 121).

HUMAnN3 maps metagenomic reads to a reference database of annotated microbial genes, returning functional pathway abundances both aggregated (summed across species) and stratified (per species). We performed differential abundance analysis on both aggregated and stratified CPMs separately using the limma package (v3.58.1) (121, 122). Before analysis, CPM values were log-transformed using *log(x + 1*) to stabilize variance. A design matrix was constructed using the formula “~ 0 + MDD + BMI + sex*age,” which includes the main effects of major depressive disorder (MDD), BMI, and an interaction between sex and age but excludes an intercept. Linear models were fitted with *limma::lmFit*, and the MDD vs. Control contrast was explicitly defined and extracted using *makeContrasts* and *contrasts.fit*, as required by the intercept-free design. Finally, statistical significance was assessed using empirical Bayes moderation via *limma::eBayes* with default settings.

The *Plotly* library (v4.10.4) was used to build the Sankey diagrams (123). Only the top 30 pathways, arranged by the adjusted *P*-value, were included. Stratified pathways (i.e., pathway::species pairs) with adjusted *P* < 0.05 (MetaCyc) or *P* < 0.1 (GOMixer) were shown, whereas taxa with higher *P*-values in the stratified data set were collapsed into a single category. This was done to more accurately represent the relative contributions of significant taxa to the total abundance of each pathway. The width of each link in the diagram is proportional to the relative contribution of the upstream node to the downstream node, specifically: (i) group to pathway links are proportional to mean abundance of each pathway in each group of participants; (ii) pathway to species links are proportional to the average contribution of each species to the pathway (from the stratified table); (iii) species nodes may connect to multiple pathways, with each link scaled independently, rather than normalized across all of a species’ contributions. Node widths in this case correspond to the sum of incoming link widths and are representative of the overall influence of a species to all the significant pathways.

## Data Availability

All the supplementary figures and tables are available at Zenodo: https://doi.org/10.5281/zenodo.16993542. The raw sequencing files were deposited at ENA under the accession code PRJEB76994. The bioinformatics pipeline used for taxonomic profiling and functional analysis is written in Nextflow (124), uses conda environments for software version control, and is prepared to run on Linux machines and Slurm HPC environments. The code is available at GitHub (https://github.com/INNOBIOME/metagen-profiler), including scripts to install all dependencies. All downstream analyses were carried out in R 4.3.3 (125). All functions developed for the analysis were included in the G4Micro R package (https://github.com/INNOBIOME/G4Micro), and the scripts containing the general workflow were included in a separate repository (https://github.com/INNOBIOME/depression_scripts). A STORMS (Strengthening The Organizing and Reporting of Microbiome Studies) (126) checklist is available at https://doi.org/10.5281/zenodo.14764853.
